# Adenocarcinoma of urachal cyst associated with pseudomyxoma peritonei masquerading as abdominal tuberculosis: A case report and review of literature

**DOI:** 10.4103/0970-1591.40626

**Published:** 2008

**Authors:** Kamran Khalid, Mohammed Sarfraz Ahmed, Muhammed Saleem Malik

**Affiliations:** Department of General Surgery, Surgical Unit I - Services Hospital, Services Institute of Medical Sciences, Lahore, Pakistan

**Keywords:** Adenocarcinoma, pseudomyxoma peritonei, urachal cyst

## Abstract

A case of 14-year-old girl is reported who presented with features of tuberculous subacute intestinal obstruction. Exploratory laparotomy revealed a urachal cyst associated with pseudomyxoma peritonei (PMP). Histopathology confirmed a moderately differentiated mucin secreting adenocarcinoma of urachal cyst associated with PMP. The adenocarcinoma of urachal cyst associated with PMP is further reviewed.

## INTRODUCTION

Urachal cysts may occur at any level in the urachal remnant and may include the entire urachus. The cyst may or may not communicate with the cavity of urinary bladder. The urachal remnants are usually lined by transitional type epithelium, but focal mucinous glandular metaplasia is often seen and may provide morphological basis for the development of intestinal-type mucinous neoplasms.[1,2] Pseudomyxoma peritonei (PMP) associated with mucinous cysts of the ovary was first reported by Wells.[3] The PMP arising from colon, pancreas, endometrium, breast, common bile duct and urachus carcinoma have been reported in the literature.[2] We report a case of mucinous cystadenocarcinoma arising in a urachal cyst associated with PMP masquerading as abdominal tuberculosis.

## CASE REPORT

**Figure 1:**
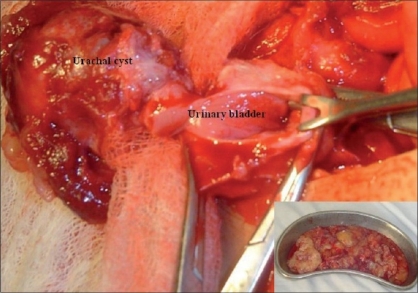
Operative photograph showing the urachal cyst being excised. The lumen of urinary bladder is open as related part of the bladder is resected along with. The inset shows myxomatous material removed from cyst and peritoneal cavity

A 14-year-old girl was admitted in December 2006 with lower abdominal pain, frequent vomiting, abdominal distension, and constipation of 6 days duration. Interrogations revealed history of recurrent abdominal pain, low-grade fever, and loss of weight during past 7 months. On examination, she was undernourished and pale. There was deep tenderness in the left lower abdomen. No viscera were palpable and bowel sounds were exaggerated. Rectal examination revealed a boggy nontender swelling in relation to anterior rectal wall. Clinical diagnosis of subacute small bowel obstruction due to abdominal tuberculosis was made. Conservative management with nasogastric decompression, intravenous fluids, and intake-output monitoring was initiated. Laboratory investigations disclosed hemoglobin 9.2 g%, white cell count 8.8 × 10^9^/L with 45% lymphocytes, ESR 86, normal renal and liver function tests, serum albumen 2.8 g%, and normal urine analysis. Her chest X-ray was normal and plain abdominal radiographs showed occasional gas fluid levels with some dilated small bowel loops. Abdominopelvic ultrasound revealed 1.6 cm gall stone in neck of gall bladder, normal pelvic organs and a 13 × 8 cm debrinous collection in the cul-de-sac. She responded well to conservative treatment and was discharged on antituberculous medications. She was readmitted in January 2007 with subacute bowel obstruction. There was tenderness and a vaguely palpable mass in the lower abdomen and bowel sounds were diminished. Her laboratory investigations were same as the first admission. Repeat ultrasound suggested a 16 × 10 cm collection seen in hypogastrium extending toward both sides of the midline and some increase in the previously reported pelvic collection (15 × 10 cm). Laparoscopy employing open technique was performed to obtain tissue diagnosis and drain the collections, but camera entered in a pool of mucoid collection. The procedure was converted to laparotomy through lower midline incision. A well-defined cyst (8 × 10 cm) filled with mucoid material was found in the extra peritoneal tissues in the midline attached to the urinary bladder. Upon opening the peritoneum, the same mucoid material was found in the pelvis and peritoneal cavity. A loop of distal ileum was stuck to the peritoneum over the posterior aspect of the cyst wall resulting in kinking, possibly causing recurrent subacute obstruction. The appendix, abdominal viscera, and pelvic organs were normal. Appendicectomy was performed and whole mucoid material was evacuated from the peritoneal cavity. The cyst and subjacent part of the dome of urinary bladder were excised [Figure 1]. Cholecystectomy was additionally performed. Her postoperative course was unremarkable. The histopathology confirmed a surgically opened, but otherwise well encapsulated cyst (10 × 16 × 12 cm) lined by tall, simple or stratified columnar epithelium continuous with foci of invasive moderately differentiated mucinous adenocarcinoma invading up to 90% of the cyst wall. No extension beyond the wall and no vascular invasion were identified. The mucoid material constituted adherent mucinous masses, positive for PAS and alcian blue stains with no detectable epithelial component consistent with PMP. The excised part of the bladder and appendix were normal and gall bladder revealed chronic cholecystitis. No communication was reported between urinary bladder and the cyst wall. Her serum tumor markers (CEA and CA 19-9) sent in the immediate postoperative period were normal. She denied any further cytoreductive procedure or adjuvant therapy and was discharged on seventh postoperative day. After a follow-up of 6 months, she is symptom free and there is no evidence of tumor recurrence on repeat abdomino-pelvic ultrasound and CT scan.

## DISCUSSION

Urachal cysts are one of the sequelae of the vestigial remnants of the embryological structure connecting allantois to the bladder apex. The cystic remnants are not uncommon and although may remain asymptomatic, they may present with mass lesions in the lower abdomen or with urinary symptoms including mucusuria. Adenocarcinomatous change in such cystic remnant accounts for 0.17-0.34% of all bladder cancers.[1–3] Gore and associates described the clinico-pathological criteria to label urachal adenocarcinoma and distinguish it from bladder or metastatic process: (1) sharp demarcation between tumor and an intact, normal surface urothelium, (2) the absence of cystitis cystica or glandularis, and (3) tumor growth in the bladder with extension in the bladder dome or anterior wall.[2] Immunohistochemically, cytokeratin 20 is expressed in more than 80% of urachal adenocarcinoma and tumor cells may express CEA and CA 19-9 with raised serum levels as tumor markers.[1,2] In 1901, Frankel reported a case in which PMP was found associated with a ruptured mucocele of the appendix.[3] The PMP is a rare and indolent disease that preferentially affects women between 50 and 70 years and appendix is now established as the primary source of most PMPs, which may then spread to other sites like ovaries.[1,2] Faulkner *et al*. reported first case of mucinous adenocarcinoma in a urachal cyst producing PMP.[3] In 1971, Mendeloff and McSwain reviewed seven adequately described cases of urachal carcinoma associated with PMP and reported their own case as eighth.[4] Local extension to the bladder was observed in most of the reported cases. Sasano and coauthors, in 1997 reported a mucinous cystadenocarcinoma in a giant urachal cyst with calcification and osseous metaplasia of the cyst wall and associated PMP.[1] No foci of rupture were detected in the cyst wall and carcinoma did not invade through the wall. They suggested that mucinous implants could result from undetected microscopic foci of carcinoma invasion through the capsule.[1] In 2004, Takeuchi and colleagues reported imaging findings of an 82-year-male operated for urachal carcinoma associated with PMP.[5] In addition, at least in some cases of urachal adenocarcinoma, elevated serum CEA and CA 19-9 provided useful information as tumor markers.[1,2] We did not have the facility for immunohistochemistry, but serum CEA and CA 19-9 were normal in our patient. Till to date 10 cases of urachal adenocarcinoma associated with PMP have been reported in the indexed literature with age ranging from 45 to 82 years.[1,3,4,5] Our case is unusual since it is observed in a 14-year-old girl presenting with features of subacute bowel obstruction. The initial diagnosis of abdominal tuberculosis was made on clinical grounds as this disease is common in this part of the world. The PMP element was initially mistaken as tuberculous peritoneal collections and the vague lower abdominal mass was considered as adherent loops of bowel and omentum frequently encountered in abdominal tuberculosis. We could have gone for more detailed imaging investigations, if abdomino-pelvic ultrasound had given us a clue of a cystic tumor in the anterior abdominal wall. Preoperative CT scan could have been a very useful tool in alerting us about the diagnosis and planning some definitive radical procedure.

To the best of our knowledge, this is the youngest age for urachal cyst adenocarcinoma associated with PMP. The mucinous adenocarcinoma found in the cystic remnant of urachus had no communication with urinary bladder and serum tumor markers were normal.
